# Genetic Regulation of Biomarkers as Stress Proxies in Dairy Cows

**DOI:** 10.3390/genes12040534

**Published:** 2021-04-06

**Authors:** Marco Milanesi, Matilde Maria Passamonti, Katia Cappelli, Andrea Minuti, Valentino Palombo, Sandy Sgorlon, Stefano Capomaccio, Mariasilvia D’Andrea, Erminio Trevisi, Bruno Stefanon, John Lewis Williams, Paolo Ajmone-Marsan

**Affiliations:** 1 Department of Animal Science, Food and Nutrition—DIANA, Università Cattolica del Sacro Cuore, 29122 Piacenza, Italy; marco.milanesi@unitus.it (M.M.); matildemaria.passamonti1@unicatt.it (M.M.P.); andrea.minuti@unicatt.it (A.M.); erminio.trevisi@unicatt.it (E.T.); johnlewis.williams@unicatt.it (J.L.W.); 2 Department for Innovation in Biological, Agro-Food and Forest Systems—DIBAF, Università della Tuscia, 01100 Viterbo, Italy; 3 Dipartimento di Medicina Veterinaria, Università degli Studi di Perugia, 06126 Perugia, Italy; katia.cappelli@unipg.it (K.C.); stefano.capomaccio@unipg.it (S.C.); 4 Dipartimento Agricoltura Ambiente e Alimenti, Università del Molise, 86100 Campobasso, Italy; valentinopalombo@alice.it (V.P.); dandrea@unimol.it (M.D.); 5 Dipartimento di Scienze Agroalimentari, Ambientali e Animali. Università degli Studi di Udine, 33100 Udine, Italy; sandy.sgorlon@uniud.it (S.S.); bruno.stefanon@uniud.it (B.S.); 6 Davies Research Centre, School of Animal and Veterinary Sciences, University of Adelaide, Adelaide, SA 5371, Australia; 7 Nutrigenomics and Proteomics Research Center-PRONUTRIGEN, Università Cattolica del Sacro Cuore, 29122 Piacenza, Italy

**Keywords:** stress, cattle, biomarkers, genetics, candidate loci

## Abstract

Stress in livestock reduces productivity and is a welfare concern. At a physiological level, stress is associated with the activation of inflammatory responses and increased levels of harmful reactive oxygen species. Biomarkers that are indicative of stress could facilitate the identification of more stress-resilient animals. We examined twenty-one metabolic, immune response, and liver function biomarkers that have been associated with stress in 416 Italian Simmental and 436 Italian Holstein cows which were genotyped for 150K SNPs. Single-SNP and haplotype-based genome-wide association studies were carried out to assess whether the variation in the levels in these biomarkers is under genetic control and to identify the genomic loci involved. Significant associations were found for the plasma levels of ceruloplasmin (*Bos taurus* chromosome 1—BTA1), paraoxonase (BTA4) and γ-glutamyl transferase (BTA17) in the individual breed analysis that coincided with the position of the genes coding for these proteins, suggesting that their expression is under cis-regulation. A meta-analysis of both breeds identified additional significant associations with paraoxonase on BTA 16 and 26. Finding genetic associations with variations in the levels of these biomarkers suggests that the selection for high or low levels of expression could be achieved rapidly. Whether the level of expression of the biomarkers correlates with the response to stressful situations has yet to be determined.

## 1. Introduction

The intensification of animal production systems has imposed a wide range of stressors on animals, including heat and metabolic stress, which are likely to increase as a result of climate change [1]. Routine management practices such as diet changes, vaccination and group rearrangement, in addition to normal physiological events, including calving, lactating and weaning, place animals under stress, particularly high-producing dairy cows [2,3]. The consequences of stress include a decrease in immune competence, with the potential for the increased occurrence of infectious diseases [4,5], and negative impacts on both production and reproductive performance [2,3,6,7,8,9,10]. Improved husbandry practices will reduce stress, but stress is inevitably associated with some aspects of livestock management. Interestingly, there are differences between individuals in the effects of stress on health [11] and behaviour. Some individuals are more prone to developing stress-related disorders than others who are insensitive or display a greater resilience to various stressors [12,13]. Studies comparing strains of mice have shown that differences in the response to acute and chronic stress has a genetic component [14]. If this could be shown for livestock, it would be possible to select animals that are genetically more likely to quickly adapt to, or recover from, stressful situations. It is essential to breed animals with good stress resilience for ethically acceptable and sustainable food production [15,16].

Responses to stress are highly complex and cannot easily be measured; therefore, identifying biological processes that act as proxy traits and finding quantifiable biomarkers for stress will accelerate the scientific understanding of stress responses and facilitate the selection for more resilient animals [17,18,19]. Stress results in the activation of the sympathetic nervous system (SNS) and the hypothalamic–pituitary–adrenal (HPA) axis, which triggers the release of catecholamines such as epinephrine, norepinephrine and glucocorticoids. The glucocorticoid cortisol has been used as a stress biomarker that is measurable in blood, milk and hair. Chronic stress impacts the immune system, increasing disease susceptibility and activating inflammatory responses [20,21,22,23,24]. Inflammation leads to changes in the serum concentrations of acute-phase proteins (APP) that are produced by the liver, and increases the production of reactive oxygen species (ROS), exposing cells to oxidative stress [25,26,27]. Dairy cows under heat stress, for example, are susceptible to oxidative stress around calving [28]. The serum concentration of some acute-phase proteins, such as haptoglobin, ceruloplasmin, serum amyloid A and globulins, increase in response to stress, while others including albumin, paraoxonase and transferrin decrease [29,30,31]. Acute-phase proteins can therefore be used as biomarkers of inflammation and oxidative stress associated with animal health and poor welfare [25].

In this study, we tested biomarkers related to metabolism, inflammation and liver function in the blood, milk and hair samples of lactating cows as genetic proxies for the stress response in two cattle breeds (Italian Holstein and Italian Simmental), which are characterised by different production performance, immune-metabolic status, selective breeding histories and genetic backgrounds [32,33,34]. Single-SNP and haplotype-based Genome-Wide Association Studies (GWAS) were run in parallel and identified SNPs that were significantly associated with the level of ceruloplasmin (CP), paraoxonase (PON) and γ-glutamyl transferase (GGT) measured in plasma. Whole genome sequences of 1059 Holstein and 156 Simmental cattle were searched to identify candidate variants controlling the variation in expression of these proteins.

## 2. Materials and Methods

### 2.1. Ethic Statement

All experimental procedures and the care of animals complied with the Italian legislation on animal ethics (DL n.116, 27/1/1992) and adhered to the bioethical rules of the University of Udine. Ethical approval for this study was given by the qualified veterinarian responsible for animal welfare of the Department of Agricultural and Environmental Science of the University of Udine. 

### 2.2. Data and Sample Collection

Blood, milk and hair samples were collected from 500 Italian Simmental (IS) and 451 Italian Holstein (IH) cows reared on 10 commercial farms in Udine province (North Eastern Italy). The animals sampled had no clinical symptoms. Sampling was organised on the day of the official milk recording. A 50 mL sample of milk was collected from each cow during the morning milking into a tube containing bronopol (Microtabs 11, Nelson-Jameson, Marshfield, WI, USA) preservative, and on the same day, hair samples were collected and stored in a paper envelope, both for the measurement of biomarkers, as described below. At the same time, blood samples were collected from the coccygeal vein during the morning milking before feeding, into two 10 mL vacutainers, one containing EDTA, for DNA extraction, and the second containing Li-heparin, for haematochemical profiling, as anticoagulants. Li-heparin samples were kept at 6–8 °C for no more than 2 hours until centrifugation at 3000 RPM for 15 min; then, plasma was collected and frozen (−80 °C) until its analysis. The local Farmer and Breeder Association [35] provided information on the animals, specifically days in milk (DIM), parity, milk yield on the day of sampling, and on management practices, including housing, ration composition, and reproductive parameters. 

### 2.3. Blood, Milk and Hair Assays

Plasma was assessed for biomarkers associated with metabolism (glucose, cholesterol, β-hydroxy-butyrate (BHB), nonesterified-fatty-acids (NEFA), creatinine and urea), innate immune response (haptoglobin, ceruloplasmin and zinc) and liver function (aspartate transaminase (AST/GOT), γ-glutamyl-transferase (GGT), total bilirubin, albumin, globulin, total protein and paraoxonase). Blood assays were performed as previously described [36] using the Instrumentation Laboratory Spa Tests (Werfen Co., Milan, Italy) on an ILAB 650 clinical auto-analyser (Instrumentation Laboratory, Lexington, MA, USA).

Milk samples were analysed for protein, fat, lactose, urea and somatic cell count (SCC) using a FT-NIR FOSS 6000 (FOSS Analytics, Hilleroed, Denmark). BHB was analysed in milk using the same test as for plasma. Cortisol, a biomarker of chronic stress, was assayed in milk and in hair, both following the procedure described by Sgorlon et al. [37].

### 2.4. Analysis of Phenotypes

The distribution of phenotypic data was tested for normality using a Shapiro–Wilk test. Non-normal phenotypes were adjusted by either the truncation of outliers (±3 SD and/or ±1.5 Q1Q3 values) or square-root transformation. The effect of factors potentially affecting phenotypic data was assessed by the analysis of variance (ANOVA) implemented in R [38], and significant factors (farm, DIM, Body Condition Score (BCS), SCC and milk yield) were included as fixed effects in the GWAS model. Animals with somatic cell counts (SCC) ≥500,000 were considered to have subclinical mastitis and were removed from the dataset. 

### 2.5. Genotyping and Quality Controls

Genomic DNA was isolated from whole blood using the GenElute Mammalian Genomic DNA Miniprep Kit (Sigma-Aldrich, Carlscad, CA, USA). A total of 951 cows were genotyped: 152 IS cows were genotyped with the BovineHD Genotyping Beadchip (Illumina, San Diego, CA, USA), containing 777,000 SNPs, and 348 IS and 451 IH cows were genotyped with the GeneSeek GGP Bovine 150K array (Neogene, Lincoln, NE, USA), containing 138,892 SNPs. SNP positions were updated to the most recent bovine reference sequence, ARS-UCD1.2 [39]. SNP markers with more than 10% of missing data, with a minor allele frequency (MAF) less than 1%, and with duplicate physical positions or that were not on autosomes were removed. Animals with more than 10% of missing data were also removed. The quality control was performed using Plink v1.9 [40,41]. 

To create a uniform set of SNP markers across individuals and breeds, the SNPs in common between the two arrays were extracted, and those present in the 150K array and absent in the HD array were imputed using BEAGLE v4.0 [42,43]. Imputation errors were identified by allelic correlation analysis (r^2^) calculated in BEAGLE. Imputed SNP markers showing an r^2^ lower than 0.75 were excluded from further analyses. After imputation, genotypes were quality-checked. Individuals with extremely low or high observed heterozygosities (ObsHet, average ± 4 SD; lower than 0.3199 or greater than 0.4414) or admixed (i.e. more than 20% of another genetic component, evaluated at K2 from Admixture software v1.3) [44]) were excluded.

Finally, SNPs with MAFs lower than 5% or Hardy–Weinberg equilibrium (HWE) *p*-values lower than 10^−6^ were removed (Supplementary Table S1). After this final filter, the dataset consisted of 350 Italian Simmental cows genotyped at 119,858 SNPs and 343 Italian Holstein cows genotyped at 121,432 SNPs.

### 2.6. Single-SNP Genome-Wide Association Study

The GWAS on all twenty-one plasma, hair and milk biomarkers, described in the Materials and Methods, were performed in GCTA v1.93.0 using the leave-one-chromosome-out (LOCO) procedure [45,46]. The regression model used for estimating the SNP marker-trait association was:

(1) Y= μ+Xb+Sa+Zu+ε

where **Y** is the vector of trait values; μ is the overall mean; **b** is the vector of fixed effects; **a** is the fixed effect of the SNP genotype; **u** and ε are vectors of random additive polygenic effects and random residuals, respectively; u~N(0, **A**σ^2^_a_) and ε~N(**0**, **I**σ^2^_ε_), where **A** is the additive genetic relationship matrix, **I** is an identity matrix, and σ^2^_a_ and σ^2^_ε_ are the additive genetic and residual error variance, respectively. **X**, **S** and **Z** are the related incidence matrices. The fixed effects included were: farm, the effect of farm; parity, the effect of the number of calvings (three levels: 1, 2 and ≥3); BCS, the fixed effect of body condition score; SCC, the covariate effect of somatic cell count, expressed as a logarithm in base 10; DIM, the covariate describing the effect of days in milk; yield, the covariate effect of milk yield. Associations were considered significant if the *p*-value was higher than Bonferroni genome-wide significant thresholds at 0.05 (from 4.28 × 10^−7^ to 4.41 × 10^−7^, based on the number of SNP markers used in the analyses). For both breeds, the suggestive *p*-value threshold was set at 10^−5^.

Heritability was calculated as the proportion of phenotypic variance explained by all SNPs. First, the restricted maximum likelihood estimation (REML) algorithm was used to estimate the variance components. Then, heritability was estimated from the ratio between additive genetic variance and phenotypic variance (VG/VP) using the GCTA suite [46]. The proportion of phenotypic variance explained (PVE) by a given SNP or haplotype was calculated following the method described by Shim et al., 2015 [47].

### 2.7. Haplotype Genome-Wide Association Study

Haplotypes were calculated and then used as if they were multiallelic genetic markers in a haplotype-based regression GWAS approach. Eagle v2.4.1 [48] was used to phase the data, and haplotypes were constructed with GHap v2.0 [49,50] using overlapping segments of four consecutive SNP markers (sliding windows moving one SNP at a time). The window size was selected based on the average inter-marker distance, which was equal to 21 kbp, and based on the calculated linkage disequilibrium, which extends up to 80 kbp in IH and 60 kbp in IS. Animals were scored 0, 1 or 2 depending on the number of copies of each haplotype allele (HapAllele) they possessed. HapAlleles were used as genetic markers in a GWAS using their allele dose. Before running the GWAS, HapAlleles were filtered, removing those with MAFs lower than 5% or Hardy–Weinberg equilibrium (HWE) *p*-values lower than 10^−6^. The same model and software (GCTA) used in the single-SNP GWAS were used in the haplotype-based GWAS. Associations were considered significant if the *p*-value was higher than Bonferroni genome-wide significance thresholds at 0.05 (from 9.16 × 10^−8^ to 9.34 × 10^−8^, based on the number of haplotypes used in the analyses). For both breeds, the suggestive *p*-value threshold was set at 10^−5^.

### 2.8. Genome-Wide Association Meta-Analysis

A meta-analysis combining the data from both breeds was run using the METAL software [51], based on the approach described by Stouffer et al. [52]. First, the direction of the effect and the *p*-value observed for all SNPs in the analysis of each breed were converted into a signed Z-score. Then, each signed Z-score was weighted by the square-root of the sample size of the two breeds before being summed. The overall Z-score was reconverted into *p*-values and thresholds were set at a nominal value of 0.05 following the Bonferroni correction for the number of SNP markers (4.71 × 10^−7^). The suggestive *p*-value threshold was set at 10^−5^, as for single-breed analyses.

### 2.9. Analysis of Candidate for Putative Causative Variants 

Variants within 1 Mbp flanking the most significant SNPs in the single-SNP GWAS were identified using the 1059 Holstein and 156 Simmental genome sequences available from the “1000 bull genome” Run 8 database [53]. Variants in high linkage disequilibrium (LD) (r^2^ > 0.3) with the SNP with the lowest GWAS *p*-value [54] were analysed for the impact on protein structure and function using the Ensembl Variant Effect Predictor (VEP) suite [55]. The effect of non-synonymous variants by the SIFT algorithm [56].

The on-line Lasagna-search 2.0 tool [57] was used to identify the transcription factor binding site (TFBS) in the 2000 bp upstream of transcription initiation sites of *CP*, *GGT1, GGT5* and *PON1*. Variants extracted from the 1000 bull genome RUN 8 dataset and intersecting TFBS were analysed for LD with the most significant SNPs identified in the GWAS. 

## 3. Results

### 3.1. Blood Assays and Outlier Analyses

A total of 78 IH and 63 IS had SCCs higher than 500,000, most likely due to subclinical mastitis, and were therefore excluded from the genomic analyses. Blood metabolic parameters recorded in all other animals were within a normal range of physiological values (Table 1) [30,58], and veterinary inspection did not identify any of these cows that had clinical symptoms or any signs of disease.

### 3.2. Single-SNP GWAS Results

The final SNP dataset comprised 343 IH individuals genotyped at 121,432 SNPs, and 350 IS cows genotyped at 119,858 SNP. The dataset used for phasing, to identify haplotypes for the GWAS, comprised 116,904 SNP for IH that identified 872,520 haplotype alleles (HapAlleles) in 120,913 haplotype blocks (HaploBlocks), and 113,592 SNPs for IS that identified 961,249 HapAlleles in 117,452 HaploBlocks. Both single-SNP and haplotype GWAS analyses identified genome-wide significant associations for only three phenotypes: GGT, CP and PON (Supplementary Tables S2 and S3). The heritabilities of these biomarkers range from 0.06 (s.e. 0.14) for CP in IH to 0.69 (s.e. 0.13) for PON in IS (Supplementary Table S1).

#### 3.2.1. Ceruloplasmin (CP)

**Figure 1:**
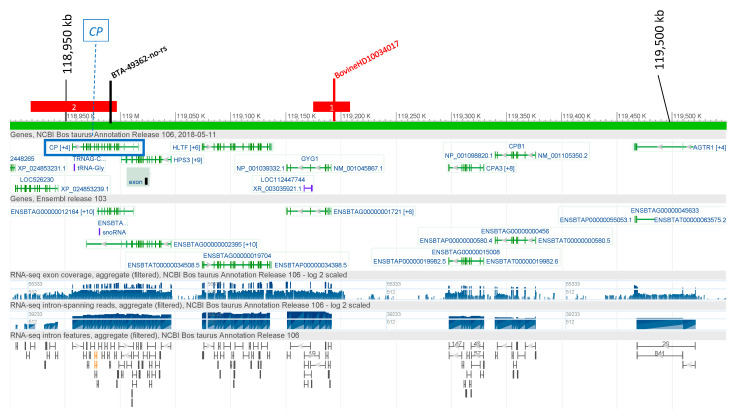
Association signals on BTA1 for ceruloplasmin. Single SNPs with SNP names are indicated, HaploBlocks are indicated as rectangles containing the number of overlapping HaploAlleles in the significant region. SNP markers and haploblocks are indicated: Black = Italian Holstein; red = Italian Simmental. The location of the *ceruloplasmin* gene (*CP*) is indicated by the blue box.

The single-SNP analysis identified a significant SNP (BTA-49362-no-rs) associated with CP in IH at 118,970 kb (Figure 1), within the *CP* gene (118,956,369–119,016,682 bp) on BTA1. In the same region, the haplotype analysis detected haplotypes reaching the genome-wide suggestive threshold (Supplementary Figure S1, Supplementary Tables S2 and S3). For IS, a single significant SNP was detected on BTA1 at 119,190 kb in the 3′ region of *CP*. Three significant HapAlleles were found in the interval 118,900–119,220 kb. The two most significant HapAlleles included the *CP* gene (see Figure 1).

#### 3.2.2. Paraoxonase (PON)

**Figure 2:**
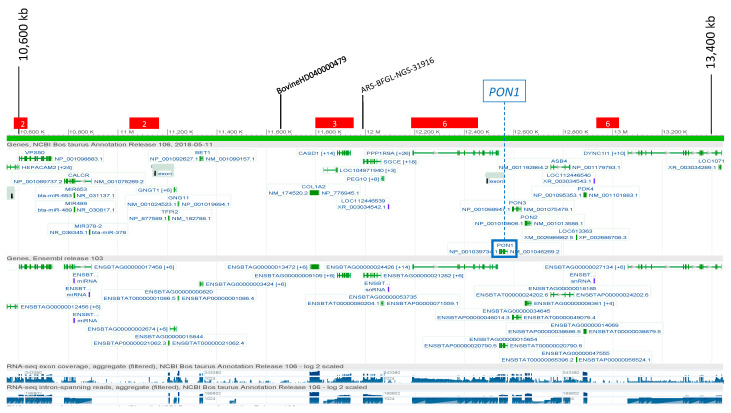
Association signals on BTA4 for paraoxonase. Single SNPs with SNP names are indicated and HaploBlocks are indicated as rectangles containing the number of overlapping HaploAlleles in the significant region. SNP markers and haploblocks are indicated: Black = Italian Holstein; red = Italian Simmental. The location of the *paroxonase-1* gene (*PON1*) is indicated in the blue box.

Two SNPs on BTA4 were significantly associated with the level of PON for IH (Figure 2), at 11,668 and 11,992 kb, which are close to *PON1* (12,542,354–12,576,328 bp). Haplotypes that were close to significance were identified in the same region but did not reach the genome-wide threshold (Supplementary Tables S2 and S3). No single SNP reached the significance threshold for IS, but a peak approaching significance was seen that overlapped the significant peak for IH. In addition, 26 significant HapAlleles were identified at nine neighbouring, non-overlapping BTA4 regions (from 10,500 to 18,900 kb, Figure 2), which were close to, but not overlapping, the *PON1* coding region. In addition to the association identified on BTA4, which included *PON1*, a suggestive signal was seen on BTA16 in both IH and IS for the single and haplotype GWAS analyses, and a second lower signal was also seen on BTA26, again at overlapping positions in both breeds (Supplementary Figure S2). These were tested further in a meta-analysis of IH and IS data (see below).

#### 3.2.3. γ Glutamyl Transferase (GGT)

**Figure 3:**
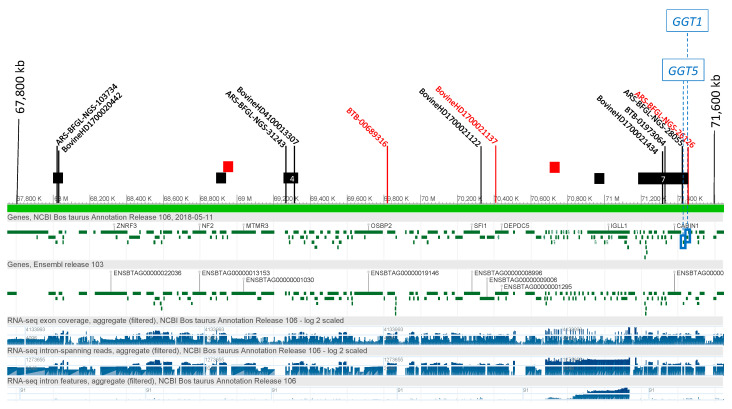
Associations on BT17 with γ-glutamyl-transferase level. Single SNPs, with SNP names, significantly associated with the GGT phenotype are indicated, and HaploBlocks are indicated as rectangles containing the number of overlapping HaploAlleles in the significant region. SNP markers and haploblocks are indicated: Black = Italian Holstein; red = Italian Simmental. The locations of the *γ-glutamyl-transferase-1* and *γ-glutamyl-transferase-5* genes (*GGT1* and *GGT5*, respectively) are indicated in the blue boxes.

Eight SNPs with genome-wide significance were associated with the level of GGT for IH on BTA17, between 67,998 and 71,462 kb (Figure 3). Seventeen significant HapAlleles were also identified at five BTA17 regions, between 68,000 and 71,300 kb near to, but not overlapping, the significant SNP. The two most significant HapAlleles (BTA17_B3319_71348361-71461810_TTTG) spanned a region that included *GGT5* (71,443–71,453 kbp) and *GGT1* (71,455–71,471 kbp). For IS, three SNPs had genome-wide significance for GGT levels, in the same BTA17 region as that detected in IH between 69,823 and 71,484 kb (Supplementary Figure S3, Supplementary Tables S2 and S3). Four HapAlleles were significantly associated with GGT levels in two regions nested within the regions identified in IH, between 68,900 and 70,500 kb, although the *GGT5* and *GGT1* genes map outside these regions. 

### 3.3. Genome-Wide Association Meta-analysis

**Figure 4:**
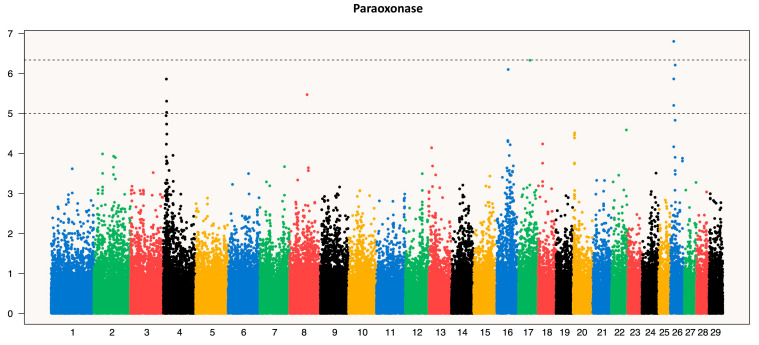
Manhattan plot of PON Genome-Wide Association Studies (GWAS) single-SNP meta-analysis. The upper horizontal dotted line is set at the nominal significant threshold values of *p* = 0.05 following Bonferroni correction for the number of SNP markers. The lower dotted line indicates the suggestive threshold value. The BTA4 peak corresponds to the PON1 gene. A significant peak is seen in BTA26 and a suggestive peak is seen in BTA16. An isolated significant SNP marker is seen in BTA17.

A meta-analysis [59] combining data from IH and IS for CP, GGT (Supplementary Figures S4 and S5) and PON (Figure 4) confirmed the significant associations identified in the single-breed GWAS in close proximity to the genes coding for these three proteins (Supplementary Table S4). Three of the five most significant SNPs associated with ceruplasmin on BTA1 (BTA-49362-no-rs) in the meta-analysis were also significant in the IH GWAS at 118,970,003 bp. The SNP BovineHD0100033931 at 118,988,242 bp and ARS-BFGL-NGS-50236 at 118,992,353 bp map within the *CP* gene (118,956,568–119,003,902). The two most significant SNPs associated with PON on BTA4 (BovineHD0400003686 at 12,507,353 bp and BovineHD0400003711 at 12,594,730 bp) closely flank the *PON1* gene (BTA4: 12,542,349–12,576,241 bp). On BTA17, the peak spanning 172 kb from ARS-BFGL-NGS-117653 (71,333,851 bp) to ARS-BFGL-NGS-74168 (71,505,929 bp) includes both *GGT5* (71,443,756–71,452,943) and *GGT1* (71,455,671–71,471,655). The most significant SNP close to these two genes was ARS-BFGL-NGS-26126 (71,483,760 bp).

In addition to the significant SNP close to *CP*, *PON1, GGT1* and *GGT5*, the significance of the suggestive signal on BTAs 16 and 26 associated with the level of PON increased. The SNP on BTA16 had an increased *p* value but did not reach genome-wide significance, while the SNP on BTA26 did reach genome-wide significance threshold in the meta-analysis. The SNP with the highest significance on BTA16 was BovineHD1600012174 at 42,784,481. There are five genes within a 500 kb interval flanking this SNP; the closest is *castor zinc finger 1* (*CASZ1*) followed by *peroxisomal biogenesis factor 14* (*PEX14*), and the other genes are *TAR DNA binding protein* (*TARDP*), *spermidine synthase* (*SRM*), *mannan binding lectin serine peptidase 2* (*MASP2*) and *exosome component 10* (*EXOSC10*). The signal on BTA26 became the most significant association for PON, higher than the SNP within the *PON1* gene. The most significant SNP on BTA26 in the meta-analysis is BovineHD2600003408, at 13,116,737, in the 5′ of the *domain containing E3 ubiquitin protein ligase 2* (*HECTD2*) gene. A second SNP near to the significant SNP, BovineHD2600003402, at 13,083,916 bp also maps within *HECTD2*. Two other genes map within 500 kb flanking this SNP: *Polycomb group ring finger 5 (PCGF5*) and *protein phosphatase 1 regulatory subunit 3C* (*PPP1R3C*). A single isolated significant SNP (BTA-113142-no-rs) on BTA17 at 42,821,258 that was above the significance threshold falls in an intergenic gene-poor region and no gene maps in the 500 kb flanking interval.

### 3.4. Candidate Causal Variant Identification

Within a 500 kb interval upstream and downstream of the most significant SNPs, a total of 1446 variants with r^2^ > 0.3 were identified in IH and 633 in IS (Supplementary Table S5). Examining 5 kb up- and downstream of *CP*, *PON* and *GGT*, 373 SNP were associated with *CP* in IH, of which 173 were within the boundaries of the gene, and in IS, 86 were associated with *CP*, of which 82 were within the boundaries of the gene. No significant SNPs were associated with *PON1* in either breed. In HS, 76 SNPs were found in *GGT1*, including the 5 kb flanking regions, of which 52 were within the gene boundaries and 33 were associated with *GGT5*, of which 16 were within the *GGT5* gene.

Of the variants within the genes, those with a moderate-to-high effect on the protein were identified using VEP. Five missense and two frameshift mutations were found in the *CP* gene in IH. Among them, a non-synonymous variant (rs43265346) that changes an asparagine into a histidine was classified as deleterious by the SIFT algorithm (SIFT score = 0.03). The other variants were classified as tolerated by SIFT. A single missense variation was identified within the *GGT1* gene boundaries in IH, which was classified as tolerated by SIFT. No variant of high or moderate effect was identified by VEP in IS within the *CP* gene.

The analysis of the *CP*, *GGT1, GGT5* and *PON1* promoter regions identified 193 variants (Supplementary Table S6). Eleven of these were in LD with the most significant SNPs in the single-SNP GWAS and were located in sites recognised by transcription factors (Table 2).

On BTA1, two variants in linkage with the most significant SNP for CP in Italian Simmental were mapped within transcription binding sites upstream of the *CP* transcription start site. One at position 118,900,034 bp (−1347 bp relative to gene transcription start site) is within a binding site recognised by both Serum Response Factor (SRF) and ETS Transcription Factor (ELK1). The second is at position 118,900,683 bp (−698 bp from the start site) within a Forkhead Box J2 (FOXJ2) binding site. The LD between these variants and the most significant SNP was above the threshold set for linkage disequilibrium (r^2^ > 0.3). In Holstein, variants in the same upstream region had a low LD (r^2^ < 0.3) with the most significant CP SNP detected in Italian Holstein.

On BTA17, five variants were in LD with the most significant SNP for GGT in IH. Four were within transcription binding sites upstream of *GGT1* at position 71,471,816 bp (−161 bp from *GGT1* start site), within an Activating Transcription Factor 1 (ATF1) binding site; at position 71,472,255 (−600 bp from the start site) in a Vitamin D Receptor Transcription Factor (VDR) binding site; at 71,472,377 bp (−722 bp from the start site), a site recognised by two transcription factors, MYC-Associated Zinc Finger Protein (MAZ) and Sp1 Transcription Factor (SP1); at position 71,473,234 bp (−1579 bp from the start site) and in a binding site for both Chicken Ovalbumin Upstream Promoter Transcription Factor 1 (COUP-TF1) and Estrogen Receptor 1 (ER-α) factors. A single variant in LD with *GGT5* was detected upstream of the gene at position 71,455,325 (−1956 bp from the transcription starting site), within a SMAD Family Member 4 (SMAD4) transcription factor binding site. These variants were in strong LD with the most significant SNP (r^2^ > 0.4) with the highest values observed for variants mapping in MAZ and SP1 (r^2^ = 0.641) and in ATF1 binding sites (r^2^ = 0.641). In IS, variants in the same upstream region had a weak LD (r^2^ < 0.3) with the most significant GGT SNP detected.

On BTA4, no variants in transcription binding sites were identified above the LD threshold (r^2^ > 0.3) with the most significant SNP for PON.

## 4. Discussion

### 4.1. GWAS Analyses

Stress is associated with various physiological and metabolic responses that differ among animals. If the predisposition of animals to higher levels of stress can be predicted from variations in the biomarkers measured under non-stressed conditions, these may be used to investigate complex stress responses and to select animals that cope better in stressful situations. The current study investigated the variation in the levels of biomarkers previously reported to differ between stressed animals and controls [30,36,60,61]. Our results showed that there is considerable variation in the levels between individuals of most of the biomarkers tested. The genetic control of expression of these biomarkers was assessed, and their genetic architecture was investigated to determine if they could act as intermediate phenotypes (“endophenotypes”) [62] that may be used to investigate stress traits, or applied directly in genomic breeding programmes to improve stress resilience in dairy cattle. 

Among the twenty-one putative stress biomarkers tested, genome-wide statistical significance in both single-SNP and haplotype-based GWAS analyses was only found for ceruloplasmin (BTA1) paraoxonase (BTA4) and γ-glutamyl transferase (BTA17). The levels of these three biomarkers had an estimated heritability between 0.06 (s.e. 0.14), for CP in IH, and 0.69 (s.e. 0.13), for PON in IS, although the small number of animals analysed does not allow an accurate estimation of heritability. In each case, a well-defined chromosomal region reached genome-wide significance, which included the gene itself (or in the case of GGT, the gene family), in both breeds analysed, the Italian Holstein and Italian Simmental. This suggests that the control of expression of these biomarkers is regulated by a variation near or close to the gene, and there are no major direct effects of other genetic loci on their expression. The most significant SNP for these biomarkers explains 7.33% and 8.87% of the phenotypic variance for CP in IH and IS, respectively; 7.98% and 6.69% for PON in IH and IS, respectively; and 14.64% and 8.08% for GGT in IH and IS, respectively. Genetic selection could rapidly change allele frequencies of these genes and, hence, the expression of the biomarker, which would be valuable if they can be shown to be associated with improved stress response.

The three biomarkers for which significant genetic associations were found are all primarily produced by the liver with functions related to protection from oxidative stress. Oxidative stress is associated with various diseases, including diabetes, atherosclerosis and cancer [63], and in the brain, where high oxygen consumption is associated with the production of free radicals; oxidative stress has been linked with a range of psychological and anxiety-related disorders in humans [64]. The ability to control the effects of oxidative stress is likely to impact the response of an individual to stressful situations. Two of the biomarkers, CP and GGT, are also associated with the acute-phase inflammatory immune response. Psychological stress can activate an inflammatory response [65]. Proinflammatory cytokines, which are mediators of the inflammatory response, communicate with the brain and affect neuroendocrine activity in the brain, inducing emotional, cognitive, and behavioural changes [66]. Indeed, prolonged exposure to chronic stress results in neurophysiological changes, including glucocorticoid resistance and changes in the cytokine response that increase the susceptibility to stress.

The *CP* gene is located on BTA1 and is approximately 60 kb long. Ceruloplasmin is an a-2 glycoprotein involved in copper transport and iron metabolism, and it is an antioxidant produced by the liver [67,68]. Levels of ceruloplasmin increase in the plasma during the acute phase of inflammation [69]. Genetically modified murine models of experimental colitis show that CP plays a role in reducing bowel inflammation [70]. In *CP* knockout mice, the impact of CP deficiency results in the accumulation of iron in the hippocampus that impacts brain neurochemistry and behaviour. The *CP* knockout mice showed elevated anxiety levels compared with normal mice [71]. This suggests that CP could be involved in reducing anxiety in stressful situations, and indeed in dairy cattle, CP has been shown to increase in relation to stress, e.g. around calving [58,72,73].

The gene encoding the acute-phase protein PON is located on BTA4 and is approximately 33 kb long. PON is a high-density lipoprotein-associated enzyme that protects lipoproteins against oxidation. *PON1* knock-out mice on a high-fat, high-cholesterol diet develop atherosclerosis more rapidly, and the LDL in their artery walls is more highly oxidised than in controls [74]. In contrast, in *PON1*-overexpressing transgenic mice, there are fewer lesions and the oxidation status of aorta is improved. Oxidative stress and the consequent lipid oxidation are thought to play a role in psychiatric disorders. In humans, decreased PON activity and specific polymorphisms in the gene have been associated with clinical conditions, including depression, generalised anxiety disorder (GAD) and schizophrenia [75]. The concentration of PON has been shown to be inversely related to oxidative stress [76] and decreases during the acute-phase response induced by stress [36], e.g. associated with calving [60,73]. Six polymorphisms in the *PON1* gene promoter region have been found to be associated with plasma PON levels in Brazilian Holstein [77].

The *GGT1* and *GGT5* genes are located on BTA17 and are approximately 22 kb long. The GGT enzyme is a biomarker of liver function. GGT is a membrane-bound glycol-protein, the production of which increases during the acute-phase response to stress-induced inflammation and oxidative stress [78]. Under conditions of oxidative stress, GGT plays a key role in the biosynthesis of glutathione (GSH), which is a potent antioxidant [79]. *GGT1* knock-out mice showed chronic GSH deficiency, growth retardation, skeletal abnormalities and cataracts, and they died younger [80]. *GGT1* is in a large linkage block containing numerous genes, including *GGT1, GGT5, GSTT1* and *GSTT*. *GGT1* knock-out mice have no glutathione activity in the spleen, small intestine and kidney, suggesting that GGT5 does not compensate for the lack of *GGT1*.

The only SNPs above the genome-wide threshold for significance in the individual-breed GWAS were those associated with *CP*, *PON1, GGT1* and *GGT5*. However, two additional signals were identified on BTAs 16 and 26 associated with the expression of PON. The signal on BTA16 was above the genome-wide suggestive, the one on BTA26 above the significant threshold in the meta-analysis, in which data from both breeds were combined in the GWAS. Indeed, the significant SNP on BTA26 had a higher *p* value than the SNP associated with PON on BTA4. The significant BTA26 SNPs are within the domain containing the E3 ubiquitin protein ligase 2 (*HECTD2*) gene, which is involved in the function of the innate immune system and is a crucial regulator of cytokine secretion [81]. The closest gene to the SNP with the highest suggestive-significance on BTA16 is castor zinc finger 1 (*CASZ1*). CASZ1 is involved in blood pressure regulation [82], neuronal development [83] and inflammation [84], all functions that are affected by stress. The next closest gene is *peroxisomal biogenesis factor 14* (*PEX14*), which also plays a role in countering oxidative stress [85]. Interestingly, *PEX14* seems to have been under selection during domestication [86], where docility and the tolerance of stress are key traits.

### 4.2. Search for Candidate Causative Variants

Significant GWAS associations were identified for the plasma levels of CP, PON and GGT. The significant SNPs in the GWAS were close to the locations of the genes coding for these proteins. Therefore, it is likely that the functional variants are close to these genes in sequences regulating their expression levels. A search of the 1000 bull sequence data identified a total of 556 variants in LD with the most significant SNP for each of the three genes, of which 457 were in *CP*, 373 in IH and 86 in IS, of which two were in common. None of the variants were above the LD threshold for *PON1*, in either breed. In IH, 76 variants were in *GGT1* and 33 in *GGT5*, most of which were within the coding region or introns (Supplementary Table S5). Among these, a single missense variant in *CP* in IH was classified as deleterious by SIFT. All other variants were classified as tolerated. Although we cannot exclude that these variants may affect the level of the proteins investigated, we focus our discussion on variants in transcription factor binding sites that are more likely to affect the expression of the three proteins.

The *CP* gene has 25 variants upstream of the coding start site that affect known transcription factor binding sites, and among these, two were identified that are in strong LD with the significant SNP, one of which, 1347 bp upstream (BTA1:118900034) overlapped Serum Response Factor (SFR) and Elk-1 binding sites. ELK-1 and SRF form a tertiary complex [87] and have several functions, including intermediate gene activation associated with neuronal development, learning and memory [88]. The second at 698 (BTA1: 118900683) is within a FOXJ2 binding site. FoxJ2 can be expressed as two isoforms, FHX.L and FHX.S, that differ in their C-terminal ends. FoxJ2 is expressed early in embryonic development, in many tissues [89].

There have been 54 variants described upstream of the *PON1* coding region, among which six have been associated with increased PON expression, e.g. in relation to calving stress [90], of which two, at 12,576,347 and 12,576,853, are in weak linkage (0.2 < r^2^ < 0.3) with BovineHD0400003479, the second most significant SNP associated with PON in the present study. The first SNP is in a GATA3 binding site. GATA3 is a transcription factor involved in the development and survival of sympathetic neurons [91]. The second SNP is within a cJUN binding site. The cJUN transcription factor activates the response to oxidative stress due to ROS produced by mitochondrial dysfunction [92]. We also identified a variant at 12,576,853 in linkage with the significant SNP in our study, which has been associated with an increase in PON expression by Silveira et al. [77]. However, we did not find this SNP associated with a transcription factor binding site. Three other SNPs have been reported associated with PON levels within *PON1* transcription binding sites in cattle [77]; however, in the present study, these were not in linkage with the SNP significant for PON. 

The *GGT1* gene has 69 variants upstream of the coding region of which eight were in LD with the significant SNP for serum GGT levels. We identified transcription factor binding sites at four of these at 71,471,816, 71,472,255, 71,472,377 and 71,473,234 bp on BTA17. The SNP at BT17:71471816 is within an activating transcription factor (AFT) binding site, the SNP at BT17:71472255 is within a Vitamin D Receptor (VDR), and the SNP at BT17:71472377 is within a Myc-associated zinc-finger protein (MAZ) binding site and a Specific protein 1 (Sp1) binding site. MAZ expression is increased in chronic inflammation [93]. Sp1 is a zinc finger transcription factor expressed in many tissues. In neuronal tissues, Sp1 and Sp3 expressions is increased in response to oxidative stress, and in rodent models, protects against neuronal loss [94]. The SNP at BTA17:71473234, which is significantly associated with GGT, is within COUP-TF1 and ER-α binding sites. COUP-TF1 is activated in response to oxidative stress, as is the ER-α transcription factor, as discussed for *PON1.*

The *GGT5* gene has 45 variants upstream of the coding region, of which three are in LD with the most significant SNP in the single-SNP GWAS. Only one (BTA17:71455325) is within a transcription binding site, which is SMAD4. Smad-dependent signalling regulates the expression of the TGF-β family of cytokines, which in turn, negatively regulates neuronal morphogenesis during brain development and plays a role in neuronal disease in humans [95,96].

## 5. Conclusions

We assessed variations in potential biomarkers of stress and the genetic control of their expression. Three biomarkers, all associated with oxidative stress, showed strong genetic associations that indicated that their expression was primarily under cis-acting control. In the case of PON, two additional genetic loci were identified that were associated with the level of PON expression. Candidate genes at these loci are also implicated in oxidative stress. In this study, the genetic association between levels of biomarkers was examined in non-stressed animals. It would be interesting to assess changes in the levels of these biomarkers in stressed animals, and in particular, if genetic variations predict the response of animals to stress, which would facilitate the selection of animals that respond better to stressful situations and hence remain healthier and more productive.

## Figures and Tables

**Table 1:** Descriptive statistics for phenotypes.

**Phenotype**	**Type**	**IH**			**IS**		
		***n***	**Mean (SD)**	**Range**	***n***	**Mean (SD)**	**Range**
Body Condition Score	Animal condition	335	2.4 (0.39)	1.05–3.75	307	3 (0.47)	1.75–4
Days in milk (DIM)	Animal condition	335	167.38 (61.82)	36–283	307	154.51 (79.03)	15–404
Somatic Cell Count	Mammary health	335	4.85 (0.42)	3.48–5.68	307	4.76 (0.47)	3.6–5.7
Milk yield	Mammary metabolism	335	37.05 (9.22)	11.9–62.5	306	26.76 (6.49)	11.2–48.9
Lactations	Animal condition	335	1.99 (1.3)	01-lug	307	2.56 (1.62)	01-set
Casein	Milk composition/	335	2.5 (0.21)	1.94–3.15	306	2.73 (0.25)	1.98–3.47
Fat (milk)	Milk composition/Mammary metabolism	335	3.57 (0.65)	1.78–5.99	306	3.75 (0.65)	1.94–7.53
Protein (milk)	Milk composition/Mammary metabolism	335	3.16 (0.28)	2.43–4.03	307	3.48 (0.34)	2.59–4.53
**Blood biomarker**	**Type**	**IH**			**IS**		
		***n***	**Mean (SD)**	**Range**	***n***	**Mean (SD)**	**Range**
Albumin	Liver function	331	37.2 (3.21)	17.25–47.44	297	37.51 (1.98)	31.59–43.2
Total bilirubin	Liver function	332	0.7 (0.56)	0.02–4.61	297	1.14 (0.75)	0.03–4.29
Total protein	Liver function	331	78.61 (7.25)	30.13–104	297	77.59 (4.55)	67.9–91.29
Globulin	Liver function/Immune response	331	41.41 (7.11)	12.88–78.06	297	40.08 (5.04)	28.99–56.54
Paraoxonase	Liver function/Lipoprotein metabolism	326	106.75 (25.99)	32.29–216.22	295	102.58 (23.74)	47.38–197.44
AST/GOT	Liver function/Protein metabolism	330	105.5 (29.64)	35.55–243.2	297	90.42 (24.45)	57.34–233.68
GGT	Liver function/ Protein metabolism	331	33.73 (12.61)	9.96–128.15	297	27.47 (6.57)	14.7–64.64
Cholesterol	Liver function/Energy metabolism	331	6.28 (1.59)	1.8–10.16	297	4.8 (1.1)	2.37–10.45
Glucose	Energy metabolism	333	3.81 (0.42)	2.66–5.06	297	3.89 (0.36)	2.98–5.1
NEFA	Energy metabolism/Lipid metabolism	333	0.13 (0.08)	0.04–0.73	297	0.11 (0.06)	0.03–0.37
BHB	Energy metabolism	327	0.72 (0.24)	0.19–1.71	294	0.7 (0.22)	0.07–1.56
Ceruloplasmin	Inflammatory response	332	2.54 (0.6)	1.13–4.74	294	2.44 (0.65)	0.71–4.14
Haptoglobin	Inflammatory response	331	0.37 (0.32)	0.02–2.24	297	0.33 (0.28)	0.03–2.35
Calcium	Mineral metabolism	127	2.61 (0.23)	1.3–2.92	0	NA (NA)	NA
Zinc	Mineral metabolism/Immune function	325	13.43 (3.19)	5.18–32.7	295	12.76 (2.87)	7.28–27.81
Creatinine	Protein metabolism/Renal function	327	87.37 (9.05)	40.58–120	295	113.13 (12.26)	77.42–158.62
Urea	Protein metabolism	331	5.34 (1.43)	2.62–10.74	297	4.65 (1.13)	1.67–8.68
**Milk biomarker**	**Type**	**IH**			**IS**		
		***n***	**Mean (SD)**	**Range**	***n***	**Mean (SD)**	**Range**
BHB	Energy metabolism	316	0.16 (0.03)	0.06–0.36	297	0.17 (0.05)	0.03–0.3
Cortisol	Immune system	313	501.71 (312.18)	41.74–1822.64	299	515.46 (263.55)	60–1539.81
Urea	Milk composition/Protein metabolism	335	22.87 (8.29)	9.4–51.4	307	19.97 (6.14)	3.65–38.3
**Hair biomarker**	**Type**	**IH**			**IS**		
		***n***	**Mean (SD)**	**Range**	***n***	**Mean (SD)**	**Range**
Cortisol	Immune system	309	3.53 (2.22)	0.45–12.5	278	3.23 (1.63)	0.84–10.66

IH = Italian Holstein, IS = Italian Simmental, NA= Not Available. Columns are: Type of phenotypes (Type), number of animals (*n*), mean and standard deviation (Mean (SD)), minimum and maximum values (Range). AST/GOT: Aspartate transaminase/glutamic oxaloacetic transaminase; BHB: β-Hydroxybutyrate; GGT: γ-Glutamyltransferase; NEFA: Nonesterified fatty acid.

**Table 2:** Candidate causative variants showing r^2^ > 0.2 with high-significance SNPs.

Gene	Chr	Pos	Alt	Ref	Distance_gene_start	TFBS.N	TFBS.name	rs	r^2^.SIM	r^2^.HOL
*CP*	1	118,900,034	T	G	1347	2	Elk-1(T00250)|SRF(T00764)	rs385773690	0.337	<0.3
*CP*	1	118,900,683	C	T	698	1	FOXJ2 (long isoform)(T04169)	rs381127256	0.320	<0.3
*PON1*	4	12,576,347	G	A	19	1	GATA-3(T00311)	rs109606244	<0.3	<0.3
*PON1*	4	12,576,418	G	T	90	1	TBP(T00794)	rs377892116	<0.3	<0.3
*PON1*	4	12,576,463	G	A	135	0		rs109953053	<0.3	<0.3
*PON1*	4	12,576,634	A	C	306	2	ER-α(T00261)|COUP-TF2(T00045)	rs110459801	<0.3	<0.3
*PON1*	4	12,576,853	T	C	525	1	c-Jun(T00133)	rs381274305	<0.3	<0.3
*PON1*	4	12,576,916	T	A	588	0		rs110270756	<0.3	<0.3
*GGT5*	17	71,454,646	T	G	1277	0		rs109325809	<0.3	0.597
*GGT5*	17	71,454,782	A	G	1413	0		rs41854700	<0.3	0.402
*GGT5*	17	71,455,325	GCCC	G	1956	1	Smad4(T04292)	rs133286128	<0.3	0.566
*GGT1*	17	71,471,816	T	G	161	1	ATF(T00051)	rs210579585	<0.3	0.641
*GGT1*	17	71,471,932	A	G	277	0		rs208460991	<0.3	0.628
*GGT1*	17	71,472,255	C	T	600	1	VDR(T00885)	rs209913616	<0.3	0.632
*GGT1*	17	71,472,377	G	C	722	2	MAZ(T00490)|Sp1(T00759)	rs210564197	<0.3	0.647
*GGT1*	17	71,472,410	C	G	755	0		rs208475328	<0.3	0.618
*GGT1*	17	71,472,863	T	C	1208	0		rs209562610	<0.3	0.541
*GGT1*	17	71,473,234	C	G	1579	2	COUP-TF1(T00149)|ER-α(T00261)	rs41854716	<0.3	0.401
*GGT1* ^1^	17	71,473,281	ACC	AC	1626	0		rs464903245	<0.3	0.434

^1^ Chr = chromosome number; Pos: Chromosome position; Alt = alternative allele; Ref = reference allele; Distance.gene.start: Distance between the variant and the gene start site; TFBS.N = Number of transcription factors binding to the variant site; TFBS.name = transcription factor name(s); rs: variant name; r^2^.SIM = linkage disequilibrium between the binding site and the most significant SNP in single-SNP GWAS in Simmental; r^2^.HOL = linkage disequilibrium between the binding site and the most significant SNP in single-SNP GWAS in Holstein.

## Data Availability

The data presented in this study are available on request from the corresponding author. The data are not publicly available, due to shared ownership with breeder associations.

## Supplementary Materials

The following are available online at https://www.mdpi.com/article/10.3390/genes12040534/s1, Figure S1: Manhattan plots showing genetic associations with the levels of ceruloplasmin. Figure S2: Manhattan plots showing genetic associations with the levels of paraoxonase. Figure S3: Manhattan plots showing genetic associations with the level of γ-glutamyl-transferase. Figure S4: Manhattan plot of CP single-SNP GWAS meta-analysis. Figure S5: Manhattan plot of GGT single-SNP GWAS meta-analysis. Table S1: Significance of fixed effects and number of animals and SNPs in the working dataset. Table S2: SNPs significantly associated with CP, PON and GGT identified by single-SNP GWAS. Table S3: Haplotypes significantly associated with CP, PON and GGT identified by haplo-GWAS. Table S4: SNPs significantly associated with CP, PON and GGT identified by single-SNP meta-GWAS. Table S5. Variants in *CP*, *PON1*, *GGT1* and *GGT5* genes linked to the most significant SNP associated with CP, PON and GGT. Table S6: Variants in the promoter regions of *CP*, *PON1*, *GGT1* and *GGT5* genes linked to the most significant SNP associated with CP, PON and GGT.
