# Supplementary material for: Genetic Regulation of Biomarkers as Stress Proxies in Dairy Cows
Source: Genes (Basel). 2021 Apr 6;12(4):534. doi: 10.3390/genes12040534 (PMC8067459; doi:10.3390/genes12040534)
Supplement: Supplementary file 1 [file genes-12-00534-s001.zip › genes-1095687-supplementary_20210330/paperstress_supplementary.figures/Suppl.figures_README.docx]

**Supplementary Figures**


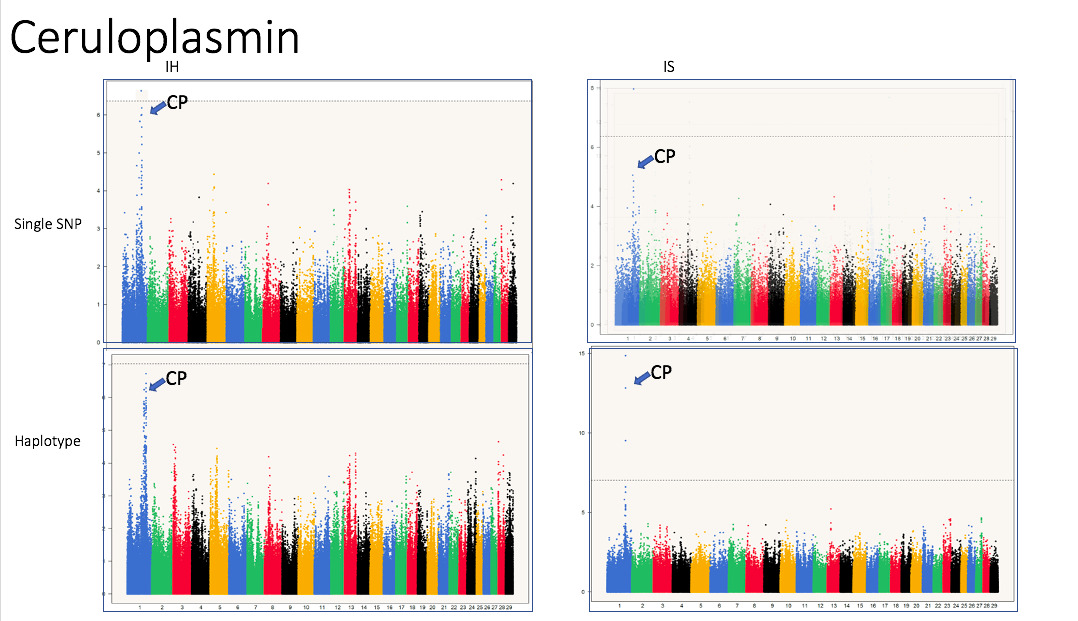


**Figure S1.** Manhattan plots showing genetic associations with the levels of ceruloplasmin. (**a**) Italian Holstein single SNP GWAS, (**b**) Italian Simmental single SNP GWAS, (**c**) Italian Holstein haplotype GWAS, (**d**) Italian Simmental haplotype GWAS. The significant SNP coinciding with the ceruloplasmin gene on chromosome 1 are indicated.


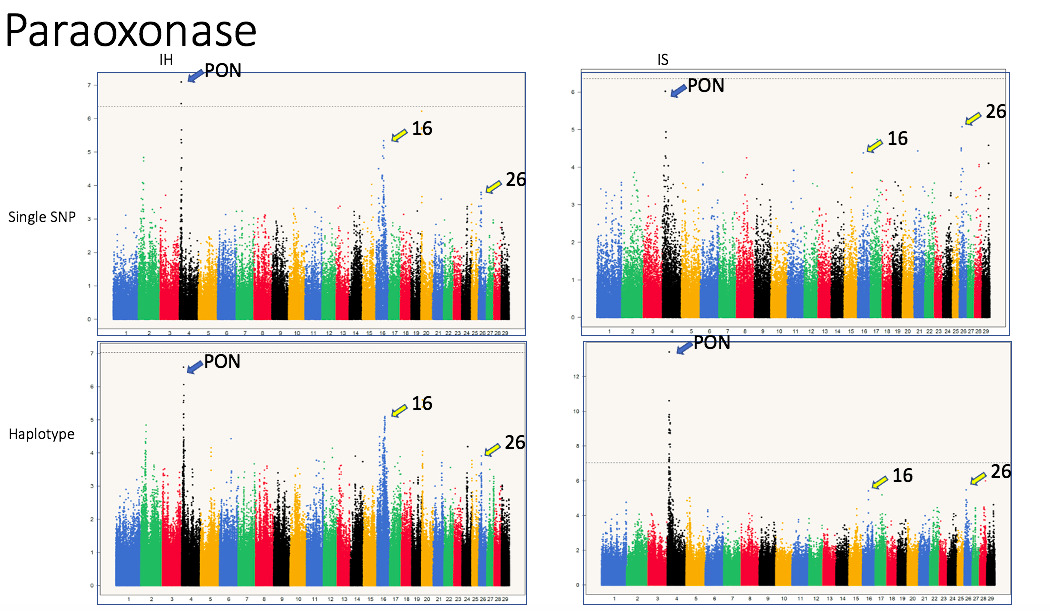


**Figure S2.** Manhattan plots showing genetic associations with the levels of paraoxonase. (**a**) Italian Holstein single SNP GWAS, (**b**) Italian Simmental single SNP GWAS, (**c**) Italian Holstein haplotype GWAS, (**d**) Italian Simmental haplotype GWAS. The significant SNP coinciding with the paraoxonase gene on chromosome 4 are indicated, and putative associations on chromosome 16 found and 26 in both breeds are also indicated.


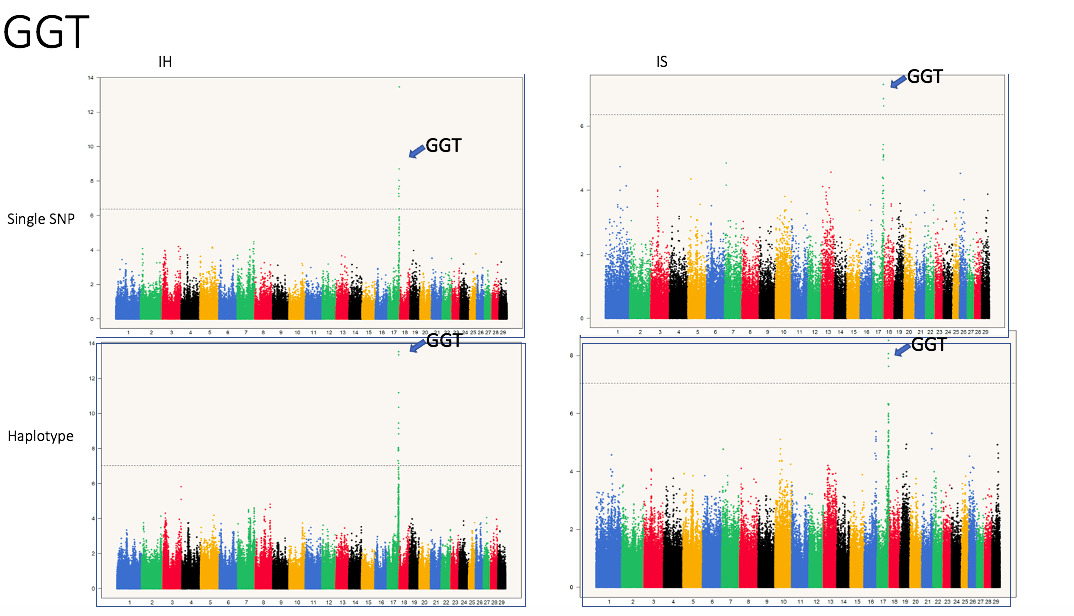


**Figure S3.** Manhattan plots showing genetic associations with the level of gamma-glutamyl-transferase. (**a**) Italian Holstein single SNP GWAS, (**b**) Italian Simmental single SNP GWAS, (**c**) Italian Holstein haplotype GWAS, (**d**) Italian Simmental haplotype GWAS. The significant SNP coinciding with the GGT gene family on BTA 17 are indicated.


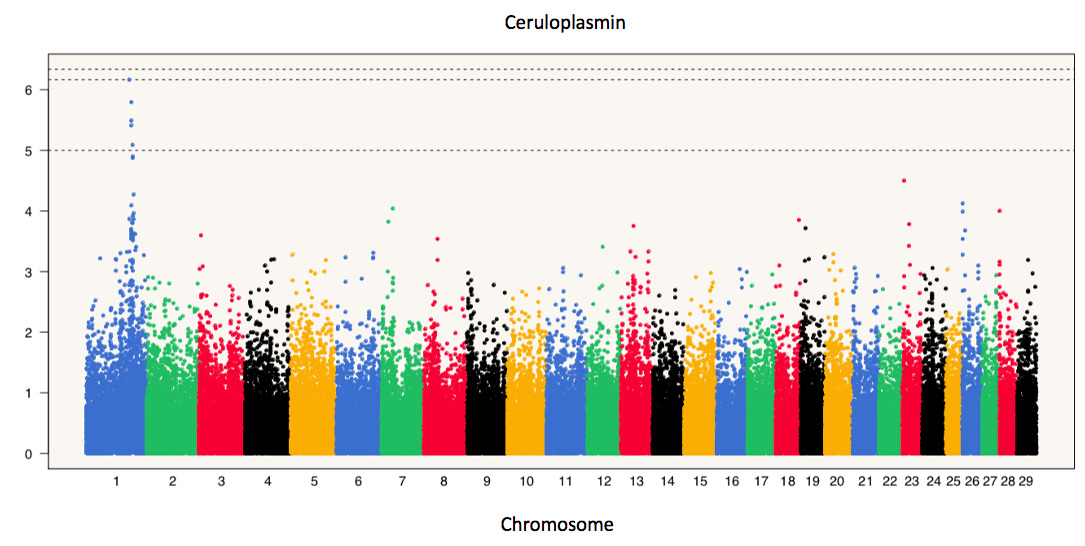


**Figure S4.** Manhattan plot of CP single-SNP GWAS meta-analysis. The BTA1 peak corresponds to CP gene.


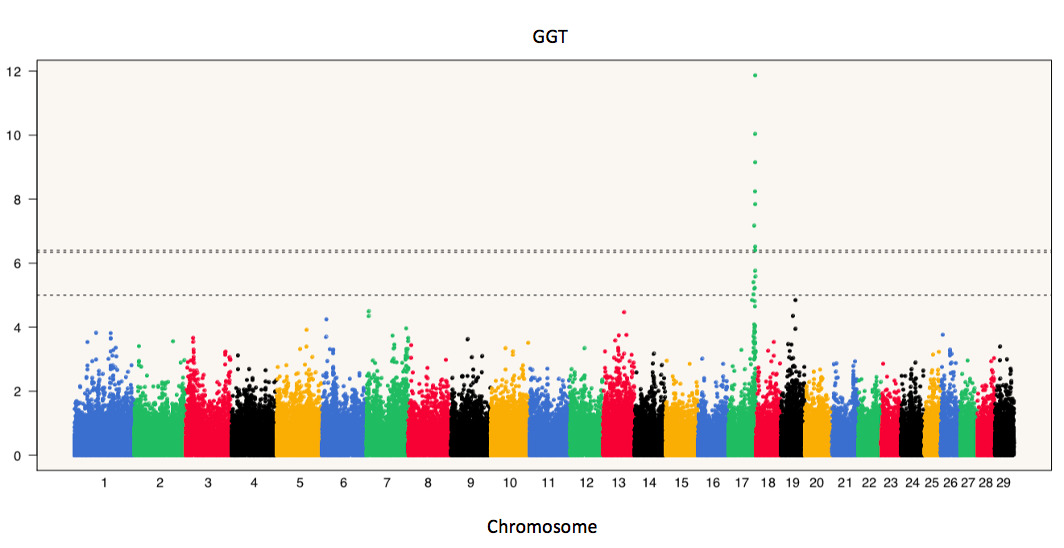


**Figure S5.** Manhattan plot of GGT single-SNP GWAS meta-analysis. *The BTA17 peak corresponds to GGT1 and GGT5 genes.*
