# Supplementary material for: Genetic Regulation of Biomarkers as Stress Proxies in Dairy Cows
Source: Genes (Basel). 2021 Apr 6;12(4):534. doi: 10.3390/genes12040534 (PMC8067459; doi:10.3390/genes12040534)
Supplement: Supplementary file 1 [file genes-12-00534-s001.zip › genes-1095687-supplementary_20210330/supplementary_tables/Suppl.tables_README.docx]

**Supplementary Tables**

**Table S1. Fixed effects’ significance and number of animals and SNPs in the working dataset.**

*IH = Italian Holstein; IS = Italian Simmental.*

*GGT(sqrt) = gamma-glutamyl transferase (root square transformation); BCS = body condition score; DIM = days in milk; LogSCC = logarithm (base 10) of somatic cell count; n. animals = number of animals in the analyses; n. SNPs (autosome) = number of SNPs in the analyses; V(G)/Vp = heritability (based on additive genetic variance); SE = standard error; P-value = significance of heritability evaluation.*

*Significance codes: 0 ‘***’ 0.001 ‘**’ 0.01 ‘*’ 0.05 ‘.’ 0.1 ‘ ’ 1*

**Table S2: SNP significantly associated to CP, PON and GGT identified by single-SNP GWAS.**

*IH = Italian Holstein; IS = Italian Simmental; CP = Ceruloplasmin; PON = paraoxonase; GGT(r2) = gamma-glutamyl transferase (root square transformation); Chr = chromosome; bp = position (in base pair); Frequency (Freq) of the reference allele (A1); SNP effect (b) of A1 allele; standard error (se); p-value (p); negative logarithm of p-value (logP); q-value (FDR); SNPs that exceeded Bonferroni significance threshold are marked with an asterisk; PVE = phenotypic variance explained.*

**Table S3: Haplotypes significantly associated to CP, PON and GGT identified by haplo GWAS.**

*IH = Italian Holstein; IS = Italian Simmental; CP = Ceruloplasmin; PON = paraoxonase; GGT(r2) = gamma-glutamyl transferase (root square transformation); Chr = chromosome; bp = position (in base pair); Frequency (Freq) of the reference allele (A1); SNP effect (b) of A1 allele; standard error (se); p-value (p); negative logarithm of p-value (logP); q-value (FDR); SNPs that exceeded Bonferroni significance threshold are marked with an asterisk; PVE = phenotypic variance explained.*

**Table S4: SNP significantly associated to CP, PON and GGT identified by single-SNP meta-GWAS***.*

*CP = Ceruloplasmin; PON = paraoxonase; GGT(r2) = gamma-glutamyl transferase (root square transformation); Chr = chromosome; bp = position (in base pair); Allele1 = reference allele; Allele2 = alternative allele; Weight = number of animals in the analysis; logMETA = logarithm (base 10) of P-value; FDR= q-value.*

**Table S5. Variants in *CP*, *PON1,* *GGT1* and *GGT5* genes linked to the most significant SNP associated to CP, PON and GGT.**

*IH = Italian Holstein; IS = Italian Simmental; CP = Ceruloplasmin; PON = paraoxonase; GGT(r2) = gamma-glutamyl transferase (root square transformation); Chr = chromosome; Pos = position (in base pair); Allele = analysed allele; Consequence = variation consequence on protein; by VEP software; SIFT = SIFT score for missense variant; Impact = consequence impact on protein; by VEP; SNP = the most significant SNP in the single SNP GWAS; r2 = Linkage Disequilibrium value between the analysed variant and the most significant SNP in the single SNP GWAS (SNP); Dist = distance (in bp) from the analysed variant and the most significant SNP in the single SNP GWAS (SNP); HOL.freq = frequency on Holstein animals from 1000 bulls dataset; SIM.freq = frequency on Simmental animals from 1000 bulls dataset.*

**Table S6: Variants in the promoter regions of *CP*; *PON1*; *GGT1* and *GGT5* genes linked to the most significant SNP associated to CP, PON and GGT.**

*CP = Ceruloplasmin; PON1 = paraoxonase 1; GGT1 = gamma-glutamyl transferase 1; GGT5 = gamma-glutamyl transferase 5; Chr = chromosome; Pos = position (in base pair); Alt = alternative allele - analysed; Ref = reference allele; TFBS.N = Number of transcription factor binding site; TFBS.name = Transcription factor binding site name.*
