# Supplementary figures and images for: Genetic Regulation of Biomarkers as Stress Proxies in Dairy Cows
Source: Genes (Basel). 2021 Apr 6;12(4):534. doi: 10.3390/genes12040534 (PMC8067459; doi:10.3390/genes12040534)

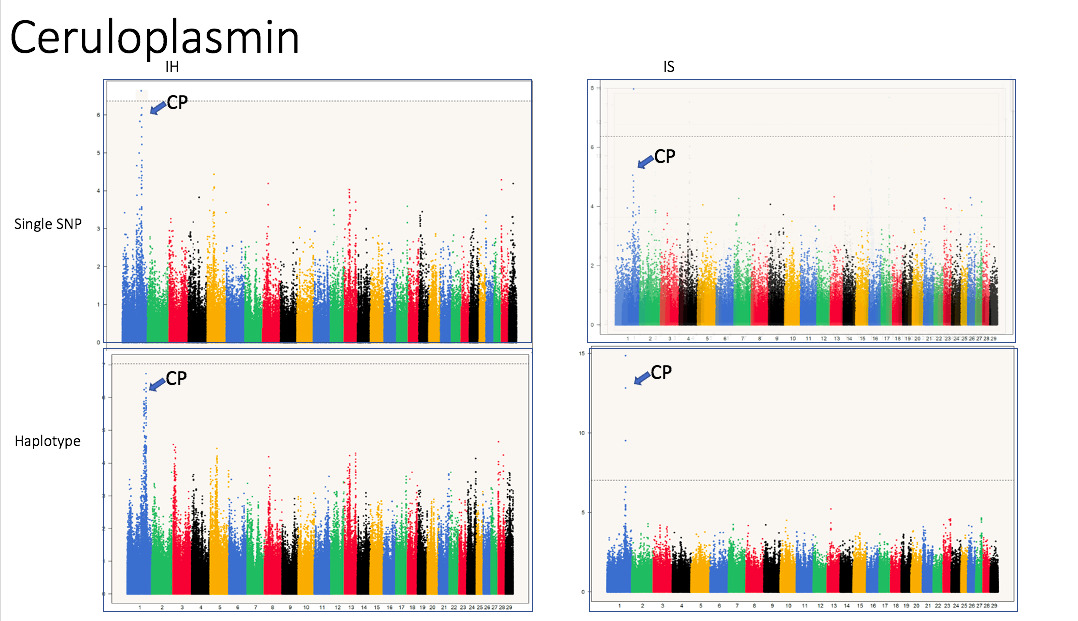

Supplement: Supplementary file 1 [file genes-12-00534-s001.zip › genes-1095687-supplementary_20210330/paperstress_supplementary.figures/Figure.S1.jpeg]

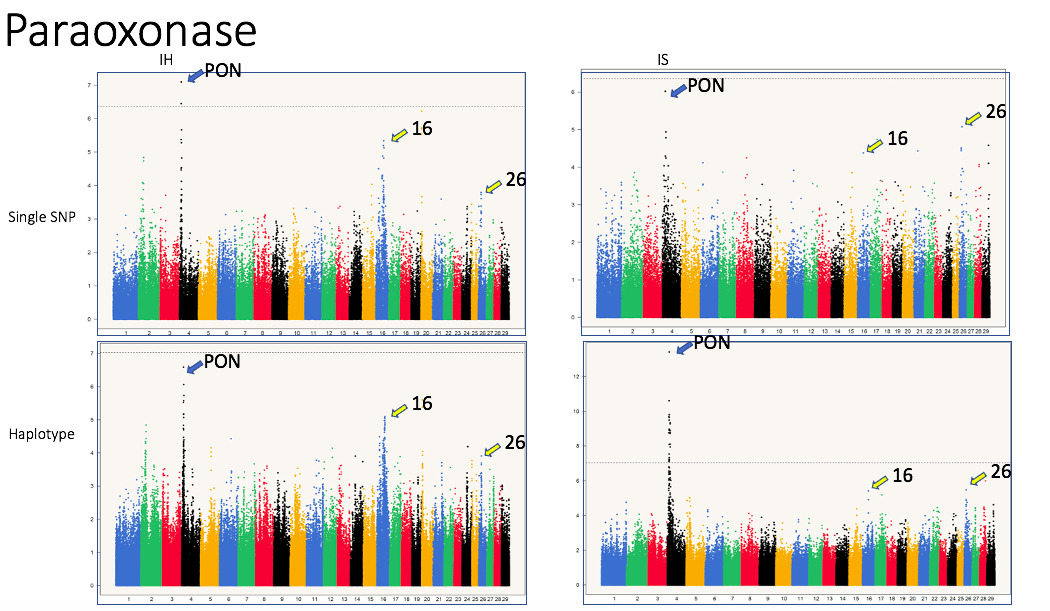

Supplement: Supplementary file 1 [file genes-12-00534-s001.zip › genes-1095687-supplementary_20210330/paperstress_supplementary.figures/Figure.S2.jpeg]

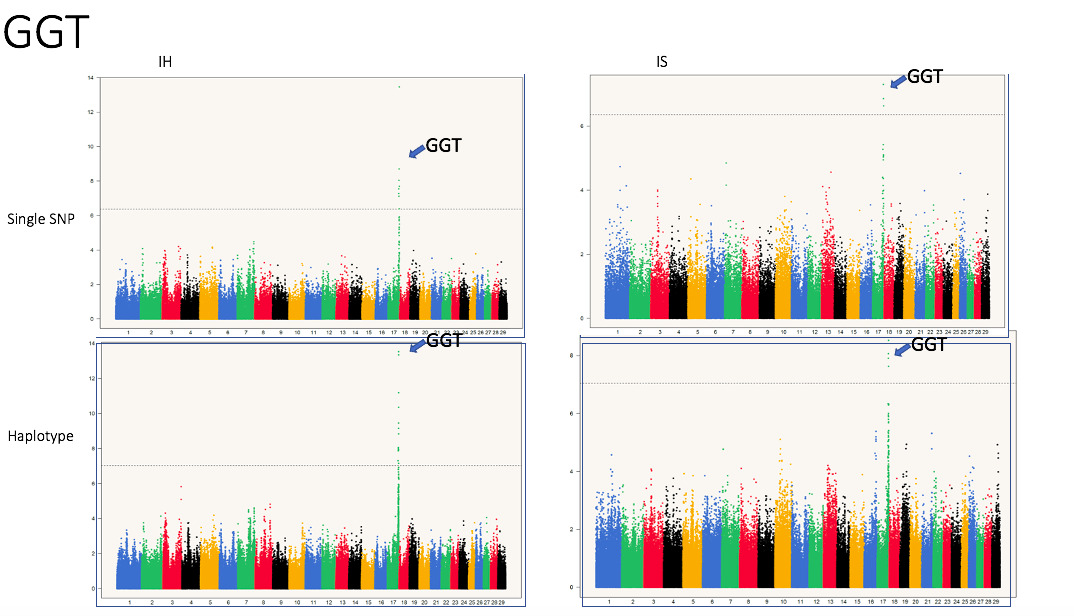

Supplement: Supplementary file 1 [file genes-12-00534-s001.zip › genes-1095687-supplementary_20210330/paperstress_supplementary.figures/Figure.S3.jpeg]

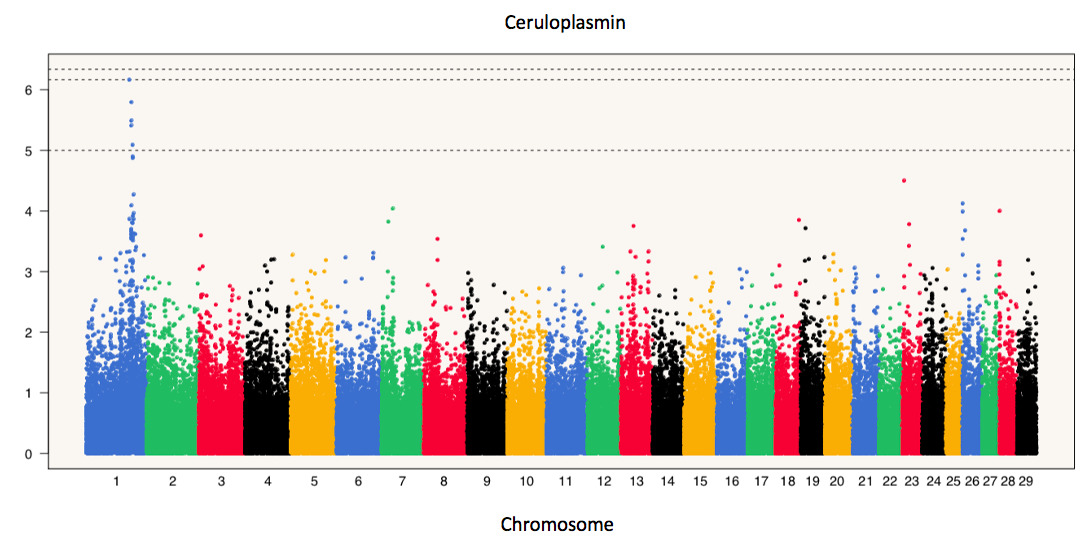

Supplement: Supplementary file 1 [file genes-12-00534-s001.zip › genes-1095687-supplementary_20210330/paperstress_supplementary.figures/Figure.S4.jpeg]

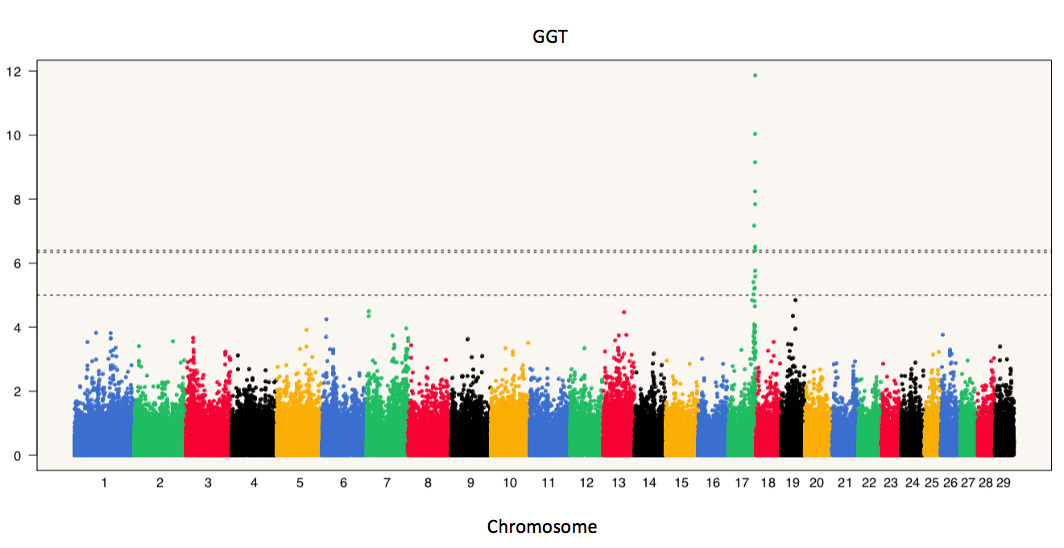

Supplement: Supplementary file 1 [file genes-12-00534-s001.zip › genes-1095687-supplementary_20210330/paperstress_supplementary.figures/Figure.S5.jpeg]
